# The Influence of Sex, Stroke and Distance on the Lactate Characteristics in High Performance Swimming

**DOI:** 10.1371/journal.pone.0077185

**Published:** 2013-10-22

**Authors:** Benjamin Holfelder, Niklas Brown, Dieter Bubeck

**Affiliations:** Department of Sport and Exercise Science, University of Stuttgart, Stuttgart, Germany; University of Granada, Spain

## Abstract

**Background:**

In order to achieve world-class performances, regular performance diagnostics is required as an essential prerequisite for guiding high performance sport. In high performance swimming, the lactate performance diagnostic is an important instrument in testing the sport specific endurance capacity. Although the role of lactate as a signaling molecule, fuel and a gluconeogenic substrate is accepted, lactate parameters are discussed concerning stability, explanatory power and interpretability.

**Methods:**

We calculated the individual anaerobic threshold (IAT) of Bunc using the swimming-specific lactate threshold test by Pansold.

**Results:**

The cross-sectional analysis (ANOVA) of n = 398 high performance swimmers showed significant effects for sex, stroke and distance on the IAT, the percentage of personal best time on the IAT (% of PB on IAT) and maximal lactate values (max. bLA). For the freestyle events the IAT decreased, % of PB on IAT and max. bLA increased from 100 to 400 m significantly in men and women. Women showed significantly higher % of PB on IAT with descriptive lower IAT in 7 of 8 analyzed events. Men showed significantly higher max. bLA in 5 of 8 events. In the second step, the analysis of 1902 data sets of these 398 athletes with a multi-level analysis (MLA) showed also significant effects for sex, swimming distance and stroke. For initial status and development over time, the effect sizes for the variables distance and sex were medium to large, whereas for stroke there were no or small effect sizes.

**Discussion:**

These significant results suggest that lactate tests in swimming specifically have to consider the lactate affecting factors sex and distance under consideration of the time period between measurements. Anthropometrical factors and the physiology of women are possible explanations for the relative better performance for lower lactate concentrations compared to men.

## Introduction

In order to achieve maximum performance in important competitions, regular performance diagnostics is required as an essential prerequisite for guiding high performance sport [1]. It is used to determine the actual performance and thus enhance the planning and periodization of the training process [2], to recognize the athletes actual stress-recovery balance and integrate it into the training schedule. For analyzing the endurance capacity in swimming, measuring lactate is common, due to the difficult conditions for spirometric testing in a pool. However, lactate parameters are currently discussed concerning their stability, explanatory power, validity and interpretability, because factors like the training state, in particular overtraining [3], diet and nutritional status [4] and the types and sizes of muscle groups and fibers [5] are affecting the individual lactate kinetics. Although research on lactate is far away from complete [1], [6] the role of lactate as a signaling molecule, fuel and a gluconeogenic substrate is accepted [5], [7], [8]. However, the determination of the individual anaerobic threshold (IAT) by means of lactate concentration is still a gold standard [9], [10]. Besides, there is currently no adequate method in swimming to substitute the lactate diagnostic in the field, thus it seems important to increase the knowledge of lactate affecting factors before, during and after exercise to further optimize the interpretation [1]. Thus, this article evaluates the use of the IAT of Bunc et al. [11] on the lactate threshold test of Pansold [12], which is used for assessing the sport specific endurance capacity in the German Swimming Association (DSV), since the beginning of the 1990 s. The choice of the threshold concept of Bunc et al. [11] can be explained by the exponential function as a common functional basis with the Pansold-test and the calculation of the IAT regarding the characteristics of the whole lactate curve. The consideration of these seems important for more reliable statements in performance diagnostics, because thereby it is easier to differentiate between sprinters, endurance athletes and untrained people [4], [13].

### Influence of Sex

Because of anthropometric, hormonal and genetic differences, sex is a major factor influencing best performances [14]. Specifically for swimming, several studies [15], [16] reported that the technique of women is more economical than the technique of men. This could be explained by anthropometrical factors like body density, a lower hydrodynamic torque and the better ability to adapt to a horizontal body alignment [15], [17]. For example, a higher body fat content, naturally observed in women [18], increases the prone gliding distance [19]. It can be assumed, that a more economical technique will cause lower lactate values of comparable load situations. However, could only identify sex-specific differences for the freestyle events, with greater post-race lactate concentrations in men. Crewther et al. [4] reported that men react to a bout of resistance training with higher lactate concentrations when compared to women. Thus, men exhibit a greater lean muscle mass and can train with heavier relative loads than women [4]. It was shown that performance differences between men and women decrease with increasing distance, also explained by physiological and morphological factors [21]. It seems that in women the aerobic metabolism and in men the anaerobic metabolism is better developed [22]. The higher content of muscle tissue and the better-developed anaerobic metabolism in men, result in higher lactate concentrations especially for 50 and 100 m events [22]. examined the muscle fiber type distribution in m. vastus lateralis of 140 healthy untrained subjects (55 women and 95 men) at the age between 19.0 and 23.9 years. No sex specific differences were found for the muscle fiber type distribution, but the area occupied by each type differs (women I>IIA>IIB; men IIA>I>IIB). In addition type IIA fibers were the largest in men, whereas type I fibers tended to be the largest in women [23]. From a physiological perspective, in an active state, glycolytic muscle fibers act as the main producers of lactate [5], [7]. In contrast, oxidative fibers serve as lactate consumers [6], also enclosing the muscle fibers of the heart within the scope of the cell-to-cell lactate shuttle [6], [7], [24].

### Influence of Stroke

Most studies about lactate in swimming were conducted in freestyle; only a few studies analyzed the influence of the other strokes on lactate. Sawka et al. [25] found similar mean lactate concentrations after 200 yd races (182,88 m) for all strokes in 23 competitive athletes. Capelli et al. [26] measured the lactate concentration after maximal swim of 50 yd, 100 yd and 200 yd in 20 male college swimmers. The descriptive data, which bases on only 3 to 8 subjects per stroke, show different orders depending on the distance. Issurin et al. [27] reported the highest lactate concentrations in butterfly, followed by breaststroke, backstroke and freestyle across three different tests with 22 highly trained swimmers (14 male, 8 females). The study of Vescovi et al. [20] with 100 swimmers (50 male and 50 females) showed significantly lower post-race lactate concentrations for breaststroke compared to butterfly and backstroke in 50 and 100 m. Regarding the four different swimming strokes it seems to be clear that freestyle followed by backstroke show the most economic energy expenditure [15], [26]. An explanation could be that freestyle and backstroke are characterized by a lower intracyclic variation of the swimming velocity compared to butterfly and breaststroke [16], [26], [28]. Butterfly and breaststroke are characterized by a gliding phase after the arm action, resulting in a greater relative loss of speed in every cycle but also underwater recoveries, especially in breaststroke [29]. A classification between butterfly and breaststroke is unclear at present [15]. Though it could be supposed, that the economy of butterfly is the slightest on account of the high technical-coordinative demand. Especially the importance of the ability to coordinate arm and leg action for a rhythmical body motion and the high demand of potential energy raising the upper body out of the water seems to be key factors for an economic technique [30]. Although, at higher swimming speeds, breaststroke seems to be less economic [15]. An explanation could be, that breaststroke is the only stroke in which great body masses are moved against the swimming direction, which means that a lot of energy will be utilized to overcome the increased drag with increasing velocity [31]. Furthermore, breaststroke is characterized by different styles of the flat and undulating technique, which influence the energy expenditure differently but also making it difficult to classify this stroke clearly [29].

### Influence of Distance

With higher swimming distance, the aerobic endurance capacity becomes more important [32]. Vescovi et al. [20] describe the post-race lactate concentrations of 50, 100, 200, 400, 800 and 1500 m events as an inverted U-shape pattern with similar concentrations for 100, 200 and 400 m. They also showed the highest post-race lactate concentrations after 200 m for backstroke and breaststroke. Similar results were shown in the study of Capelli [26], where the highest values were achieved in 200 yd for three of four strokes. From a physiological perspective, muscular power is highly determined by the muscle fiber type distribution [33]. The velocity and strength development of a muscle fiber is associated with the myosin heavy chain (MyHC) isoforms [34]. A higher content of type II fibers causes a bigger strength development [33], [35], which is essential for sprinters. Because type IIA fibers act as main producers of lactate in an active state [5], [7], an increased IAT is to be observed in sprinters with a larger amount of type II fibers. In contrast, it is supposed that longer distances require a training contribution with the trend towards achieving a maximum aerobic capacity with a greater content of type I fibers. Thus, higher training extents in low mean intensities are recommended, promoting a fiber shift towards the slower type I fibers. Type I fibers influence the lactate clearance positively [6]. This also explains, why with a higher endurance capacity the lactate curve shifts to the right [35]. Nevertheless, the right shift alone does not necessarily implicate an improvement in aerobic metabolism [1]. At a cellular level, the mitochondrial biogenesis seems to be important concerning the muscle fiber differentiation [36]. In oxidative fibers, the mitochondria can occupy 20–40% of the cell volume, whereas in glycolytic fibers only down to 1% of the cell volume is filled by mitochondria [37]. Within the scope of the intracellular lactate shuttle hypothesis, mitochondria have an important function for the lactate metabolism [7], [24]. It has to be added, that the classification of the MyHC does not completely correlate with the oxidative capacity [38]. The work of supports this impression with swimming taking a special position. The overlappings at molecular and cellular level are reflected in competitions, with some athletes achieving world-class performances in several disciplines (e.g. 100–400 m swimming) with different performance profiles. To current knowledge, specific training has to be planned for each discipline, avoiding endurance and strength specific signaling pathways overlapping and thus reducing or even eliminating training effects [38], [40], [41]. Summarized, to understand the physiological adaptations as a result of specific training content seems to be very important for interpreting lactate tests [1].

### Aims of the Study

The first aim of this article is to improve the interpretability of lactate diagnostics in swimming by identifying lactate-affecting variables. Hence, the influence of sex, distance and swimming stroke on the IAT, percentage of personal best time on IAT (% of PB on IAT) and maximum lactate (max. bLA) concentration is evaluated. The second aim of this study is to present the Multi-Level Analysis (MLA) as a statistical method which is able to analyze the typical data structure in high performance sports in a formally correct way.

## Materials and Methods

### Subjects

This investigation is based on a retrospective analysis of lactate tests from the data pool of the Olympic Training Centre Hamburg/Schleswig Holstein (GER), Department of Training Science. Because the whole data set was collected by the Olympic Training Centre Hamburg/Schleswig Holstein, a unified data collection and assessment of qualified personnel is assumed. The 18-year data-collection period itself was not monitored, thus the exact procedure of data acquisition cannot be described here. In the analysis, 2063 data sets measured between 1992 and 2010 were examined. 1902 data sets of 398 athletes [female n = 170 (42.7%, age 16.94±2.78 years, age range 13–26 years), male n = 228 (57.3%, age 19.10±3.17 years, age range = 15–36 years) met the inclusion criteria. As an inclusion criterion, only data sets of athletes with personal bests around 700 points of the LEN point table (1000 points = world record for the period of validity) were included. Furthermore only data sets were examined in which the coefficients of determination were r^2^≥0.92 (5 steps) or r^2^≥0.95 (4 steps) [42]. The data were collected as part of the regular performance diagnostics of the swimming association. After consultation of the Ethics Committee of the University Tübingen Medical School (GER), the retrospective and anonymous analysis of data of own patients, which where collected as part of diagnostics, therapy or therapy control need no guidance after the Professional Code for Physicians in Germany (§15 (1)) and no informed consent of the patients. There are no concerns of the commission about collecting, processing and publishing such data.

### Test Protocols

#### Lactate threshold test by pansold

The lactate test by Pansold [12], [43] is a swimming-specific field test for diagnosing the endurance capacity, accounting for the different structures of swimming disciplines. This test protocol is used in the DSV since the beginning of the 1990 s. The test is carried out for 100 and 200 m disciplines in five steps, for 400 m in four steps in a 50 m pool, usually in the athletes’ main event. The load specification is determined by a percentage of the individual best, whereby the rest periods between the steps are fixed [42] (cf. table 1). The step duration is reduced from step to step, because of the constant distance and increasing swimming speed. After every step the lactate concentration of the capillary blood is measured with blood samples from the ear lobe. The lactate concentration of the last step (maximum speed) represents the highest value of the measurements after 4, 7 and 10 min. Therefore this lactate concentration represents the maximum individual lactate concentration of the test. The analysis of the lactate kinetic is based on the exponential function y = a*e^(b*x)^ [y = lactate in mmol*L^−1^; a = free coefficient; b = slope coefficient, x = speed in m/s]. For the calculation of the regression coefficients “a” and “b” a quasilinear regression analysis (method of the smallest squares) is carried out. In addition, the coefficient of determination is calculated, which provides information about the reliability of the Pansold-test. With five steps r^2^≥0.92 (100 m and 200 m disciplines) and with four steps r^2^≥0.95 (400 m disciplines) [42]. The parameters of the Pansold-function were calculated with MS Access 2007.

**Table 1:** Information about the Pansold test protocol [42] (p. 169).

distance	number of steps	number of repititions	stroke	recommended intensity for the first stepin % of personal best time		break between repititions	break betweensteps	lactate measurement
				men	women			
100 m	1	3	Bu	60–65	70–75	1 min	3 min	directly
	2	2	Ba	70–75	75–80	1 min	3 min	directly
	3	1	Br	70–75	80–85		5 min	after 1 min
	4	1	Fr	65–70	70–75		approx. 20 min	after 1–3 min
	5	1	increase of 3–4 s/per steplast step = maximum speed					after 4, 7 and 10 min
200 m	1	3	Bu	70–75	75–80	1 min	3 min	directly
	2	2	Ba	75–80	80–85	1 min	3 min	directly
	3	1	Br	75–80	83–87		5 min	after 1 min
	4	1	Fr	75–80	80–85		approx. 20 min	after 1–3. min
	5	1	increase of 5–8 s/step. last step = maximum speed					after 4, 7 and 10 min
400 m	1	1	Fr	80–85	85–90	3 min		after 1 min
	2	1	increase of 8–12 s/step. last step = maximum speed			5 min		after 3 min
	3	1				up to 30 min		after 3 min
	4	1						after 4, 7 and 10 min

Abbreviations: Ba = Backstroke, Br = Breaststroke, Bu = Butterfly, Fr = Freestyle.

### The Individual Threshold Concept of Bunc et al. (1985)

**Figure 1:**
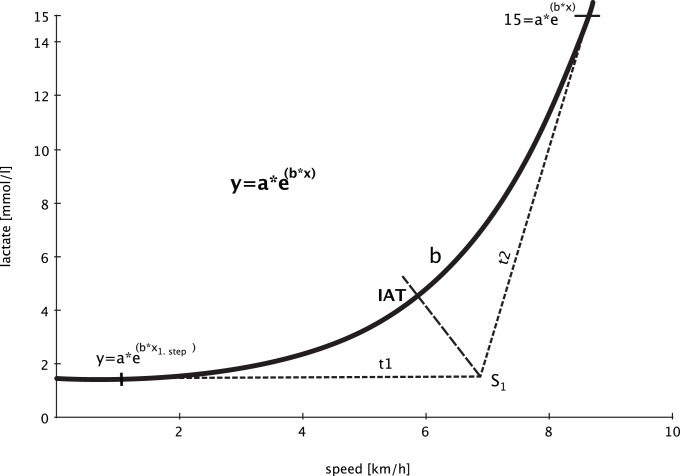
Graphical Determination of the IAT by Bunc et al. [11] with the Pansold-function y = a*e^(b*x)^ [42], [43].

The IAT of Bunc et al. [11] is represented by the point in which the inclination of the lactate-load function changes the most. Faude et al. [44] report in a recent review, that there is a high correlation between the IAT of Bunc et al. and MLSS of r = 0.98–0.99 in 16 healthy male runners and r = 0.89 in n = 22 healthy cyclists. In both cases, the running speed and the power output in watt at IAT are higher than at MLSS (+0,14–0,31 m/s/+71,5 W). For the calculation of the IAT, based on the lactate curve and the given exponential function, the following steps are recommended (cf. figure 1):

1. Tangent t_1_ to the point with the lowest load (y = a*e^(b*x^_1.step_^)^) and tangent t_2_ to the point of 15 mmol/l (15 = a*e^(b*x)^)
2. Calculate intersection (S_1_) of both tangents
3. Angle bisector by the intersection of the tangents
4. Intersection between angle bisector and lactate curve (y = a*e^(b*x)^) represents the IAT of Bunc et al. [11].

The use of this threshold concept on the exponential function of the Pansold-test y = a*e^(b*x)^ requires the extension of the Pansold-function with the lactate concentration of the first step (L_1. step_). This corresponds to the point of the lowest load in which the tangent t_1_ is calculated. The angle bisector is described by the graphical bisector with an axis relation between x-axis (km/h) to y-axis (lactate in mmol/l) from 1∶2 [11]. The extrapolation of the IAT was carried out with MATLAB®.

### Statistic Analysis

All statistics were performed using SPSS (version 19.0 for Macintosh). For analyzing the influence of sex, swimming stroke and distance on the IAT, the percentage of the personal best time on IAT (% of PB on IAT) and on maximum lactate value (max. bLA), a three-factor analysis of variance (ANOVA) was conducted for each variable for 398 subjects. Because the correlations between the three variables were only weak, three ANOVAs were calculated instead of a MANOVA. The post hoc tests of Bonferroni (equal variances), Tamhane-T2 (unequal variances) were carried out for sex, stroke and distance specific analysis to test which means are significantly different from each other. T-Tests for two independent samples were used to evaluate differences in sex in every stroke and every distance. For the athletes with more than one data set, the first data set was used for the ANOVAs, representing the initial status for the second part of the analysis.

In the second part of the statistic analysis all 1902 data sets, which met the inclusion criteria, were involved. The number of data sets for each event and sex are in table 2. Most athletes had several data sets with different in-between time intervals, therefore a Multi-Level Analysis (MLA) for IAT (Model 1), % of PB on IAT (Model 2) and max. bLA (Model 3) as the dependent variable was conducted. The advantage of this method is, that information about the change over time of the dependent variables under consideration of the in-between time intervals between measurements is given. Also valid data does not have to be excluded. Therefore, this method is more flexible and formally correct for such data structures. The comparison of the basic models (without predictors; df = 1) (A) random intercept and (B) random intercept & random slope with the help of the information criteria proved a significantly (p<.001) different change of the dependent variables of the athletes over time. Random intercept (A) is based on the assumptions that the subjects have different base values, but the same rate of change over time. Random intercept & random slope (B) assumes different base values and different change rates over time. Comparing these two basic models by using information criteria helps to fit the most economical model with the highest statistical power. “The smaller the values of these criteria, the better the fit of the model” [45] (p. 218). Values of the information criteria are difficult to interpret, but differences larger than 10 are substantial [46]. There is a decrease in the information criteria from Model (A) to (B) for Log-Likelihood (-2logL), Akaike information criterion (AIC), and Bayesian information criterion (BIC) for all dependent variables >24. In addition model (B) reduces the variance by 5.7% for IAT, by 23.48% for % of PB on IAT and by 5.6% for max. bLA (level 1-pseudo R^2^) compared to model (A), which is why further analysis are conducted with model (B). At first, the model was calculated in each case individually with the time invariant predictors (fixed) sex (S), swimming distance (D) and swimming stroke (St) to check the explained between-individual variation for each predictor on the initial status of the IAT (level 2-pseudo R^2^_C_) and the individual variation ( = slopes) of the dependent variables over time (level 2-pseudo R^2^_S_) [46], [47]. According to guideline and suggested in Kwok et al. [34], statements about the effect size can be made with the help of R^2^ changes (.02; .13; .26 representing a small, medium and large effect). Finally the following model with all three predictors for each dependent variable separately was calculated.
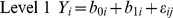
(1)

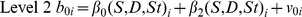
(2)

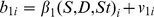
(3)


**Table 2:** Attribution of the 1902 data sets of n = 398 athletes for the multi level analysis (MLA) for each event and sex.

	100 m Bu	100 m Ba	200 m Ba	100 m Br	200 m Br	100 m Fr	200 m Fr	400 m Fr
men	66	97	120	124	78	194	347	119
women	19	66	67	75	76	145	232	77

Abbreviations: Ba = Backstroke, Br = Breaststroke, Bu = Butterfly, Fr = Freestyle.

Integrated formula:
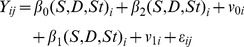
(4)


Level 1 represents athletes IAT/% of PB on IAT/max. bLA at different measuring times. Level 2 represents the different subjects (n = 398) taking the fixed predictors sex (S), distance (D) and stroke (St) into account. The alpha level of the tests was set to p<0.05.

## Results

### ANOVAs

In all three ANOVAs no significant interaction effects were found. IAT: The ANOVA with IAT [mmol*L^−1^] as dependent variable showed significant effects for sex *F*(1, 382) = 7.88, p = .005, par. η^2^ = .020, distance *F*(2, 382) = 10.98, p<.001, par. η^2^ = .054 and stroke *F*(3, 382) = 6.76, p<.001, par. η^2^ = .050. Overall, 100 m differed significantly from 200 and 400 m (p<.001). Butterfly differed significantly from all other strokes (p<.001). The descriptive data showed lower lactate concentrations on IAT for women in all events, with only 200 m freestyle being significant (p = .05; cf. table 3). Sex specific analysis for stroke per distance showed significant differences for butterfly and breaststroke (p<.001), freestyle (p = .002) and tending to be significant for backstroke (p = .076) in the males 100 m events. The highest mean values were achieved in butterfly, followed by backstroke, freestyle and breaststroke. The sex specific analysis for stroke showed significant differences (p<.001) between 100 and 400 m, as well as between 200 and 400 m freestyle, each for men and women.

**Table 3:** Differences in IAT of Bunc et[11] [mmol*L^−1^] between sexes given as means (M), standard deviations (SD) and confidence intervals of the lactate step test of Pansold and the percentage of the personal best time on the IAT (% of PB on IAT) for male (M) and female (F) of n = 398 athletes.

			IAT [mmol*L^−1^]				% of PB on IAT			
				95% Confidence Interval				95% Confidence Interval		
Event	sex	n	M ± SD	Lower Bound	Upper Bound	p	M ± SD	Lower Bound	Upper Bound	p
100 m Bu	M	15	7.52±1.69	6.70	8.59	.35	86.39±4.92	83.88	89.04	.87
	F	6	6.78±1.35	5.63	7.89		86.06±1.92	84.18	87.40	
100 m Ba	M	15	6.43±1.25	5.82	7.09	.22	87.26±2.81	85.72	88.64	.02*
	F	13	5.87±1.07	5.29	6.52		89.96±2.81	88.54	91.57	
200 m Ba	M	19	5.66±1.12	5.19	6.22	.26	88.26±1.69	87.48	89.01	.001**
	F	16	5.16±1.48	4.39	5.89		91.60±3.48	90.19	93.67	
100 m Br	M	29	5.96±1.09	5.58	6.39	.19	85.67±3.40	84.42	86.88	.02*
	F	13	5.44±1.31	4.76	6.14		88.36±3.54	86.40	90.18	
200 m Br	M	20	5.64±0.87	5.29	6.03	.06	86.93±2.94	85.66	88.22	<.001***
	F	18	5.13±0.74	4.81	5.48		91.26±1.74	90.40	92.15	
100 m Fr	M	44	6.19±0.99	5.92	6.49	.06	84.93±2.83	84.14	85.80	<.001***
	F	35	5.76±1.01	5.43	6.10		88.32±2.61	87.46	89.17	
200 m Fr	M	58	5.70±0.95	5.46	5.96	.05*	87.96±2.61	87.32	88.70	<.001***
	F	50	5.33±1.04	5.05	5.63		91.39±2.26	87.46	89.17	
400 m Fr	M	28	5.19±1.13	4.77	5.63	.96	92.56±1.67	91.90	93.20	.001**
	F	19	5.21±1.16	4.66	5.74		94.61±2.41	93,52	95.73	

p = .05*, p = .001**, p<.001***.

Abbreviations: Ba = Backstroke, Br = Breaststroke, Bu = Butterfly, Fr = Freestyle.

#### % of PB on IAT/% of PB

The ANOVA with % of PB on IAT as dependent variable showed significant effects for sex *F*(1, 382) = 40.58, p<.001, par. η^2^ = .096, distance *F*(2, 382) = 88.36, p<.001, par. η^2^ = .316 and stroke *F*(3, 382) = 3.60, p = .014, par. η^2^ = .028. Overall, 100 m differed significantly from 200 and 400 m, as well as 200 from 400 m (p<.001) with higher values in longer distances. Significant differences were found between butterfly and backstroke/freestyle (p<.001), backstroke and breaststroke (p = .006), as well as between freestyle and breaststroke (p<.001). Under consideration of the mean values and confidence intervals (cf. table 3) the lowest values were produced in butterfly, the highest for the freestyle events. Women showed significantly higher % of PB on IAT (t-test) with descriptive lower IAT in 7 of 8 events. There was also a significant difference (p = .039) for % of PB on IAT between 100 m butterfly and backstroke in women, with lower mean values in the butterfly event. For both sexes, the means are significantly higher (p<.001) with increasing distance for the freestyle events. The same effect occurred between 100 and 200 m breaststroke (p = .015) in women. The descriptive data showed (cf. table 4) that women achieve a higher % of PB with lower bLA compared to men in nearly all submaximal steps. In the last step, the % of PB values differ significant between men and women only for 200 m breaststroke (p = .041).

**Table 4:** Blood lactate concentrations (bLA [mmol*L^−1^]) and percentage of individual best time (% of PB) for each step.

		step 1		step 2		step 3		step 4		step 5	
event	sex	bLA[mmol*L^−1^]	% of PB	bLA[mmol*L^−1^]	% of PB	bLA[mmol*L^−1^]	% of PB	bLA[mmol*L^−1^]	% of PB	bLA[mmol*L^−1^]	% of PB
100 vm Bu	M	3.98±2.21	72.81±4.10	4.98±2.61	76.91±2.96	6.25±2.60	82.07±3.41	8.14±2.63	86.90±3.60	10.21±2.11	93.60±2.37
	F	3.45±1.49	73.65±4.70	4.60±1.52	78.90±1.83	6.03±1.67	83.88±1.09	7.80±1.86	88.76±1.72	10.35±2.23	93.40±2.56
100 m Ba	M	2.99±1.29	76.61±3.63	4.18±1.52	80.55±3.38	5.50±1.75	84.91±2.62	7.66±1.64	89.30±2.69	12.01±2.41	95.67±2.46
	F	2.46±0.96	80.61±3.55	3.91±1.60	84.58±4.67	5.59±2.51	86.18±4.57	7.98±3.39	88.24±5.23	11.24±3.41	95.64±2.56
200 m Ba	M	2.23±1.08	80.11±1.91	3.21±1.46	83.31±1.88	4.67±1.84	86.60±1.91	6.96±2.10	89.64±1.79	11.24±2.21	93.70±2.21
	F	1.99±1.14	83.41±2.36	2.69±1.45	85.90±2.30	3.64±1.75	88.62±2.70	5.53±2.40	91.40±2.66	9.07±3.74	94.57±2.16
100 m Br	M	2.54±1.01	74.58±3.72	3.70±1.40	78.40±2.60	5.07±1.77	82.85±2.45	7.36±2.30	86.82±2.96	10.30±2.35	92.90±2.72
	F	2.16±1.06	78.99±2.75	2.94±1.18	81.72±2.58	4.39±1.43	85.59±2.90	6.69±2.01	89.56±3.54	9.08±2.54	93.21±3.82
200 m Br	M	2.24±0.77	79.09±3.11	3.21±1.10	82.14±3.35	4.59±1.65	85.21±3.26	6.78±2.07	88.02±3.16	10.20±1.76	91.76±2.84
	F	1.84±0.57	84.80±1.97	2.86±0.86	87.34±2.04	4.20±1.15	89.79±2.04	5.78±1.34	91.64±1.75	7.99±2.10	93.81±1.73
100 m Fr	M	2.70±1.01	72.46±3.49	3.89±1.34	77.29±3.43	5.15±1.78	82.66±3.70	7.99±2.55	87.78±3.61	12.65±2.58	95.19±2.44
	F	2.35±1.01	75.24±4.36	3.52±1.29	81.77±3.32	4.84±1.64	85.96±3.24	7.23±1.93	90.07±3.40	10.82±2.26	95.40±2.53
200 m Fr	M	2.30±0.85	79.39±2.90	3.31±1.10	82.80±2.44	4.68±1.36	86.44±2.36	6.93±2.14	89.55±2.68	10.97±2.46	94.50±2.61
	F	2.03±0.85	83,.5±2.78	2.98±1.27	86.28±2.71	4.13±1.63	89.33±2.74	6.01±2.10	91.65±2.49	9.43±2.61	95.28±2.32
400 m Fr	M	1.95±0.93	85.51±2.29	2.93±1.16	88.54±2.29	4.55±1.28	91.26±2.26	8.20±1.92	95,53±2,66		
	F	1.96±0.89	87.36±1.67	2.76±1.06	90.15±2.05	4.20±1.79	94.94±2.52	6.08±2.19	94,95±2,52		

Abbreviations: Ba = Backstroke, Br = Breaststroke, Bu = Butterfly, Fr = Freestyle.

#### Max. bLA

The ANOVA with max. bLA [mmol*L^−1^] as dependent variable offered significant effects for sex *F*(1, 382) = 17.74, p<.001, par. η^2^ = .044, distance *F*(2, 382) = 49.43, p<.001, par. η^2^ = .206 and stroke *F*(3, 382) = 8.36, p<.001, par. η^2^ = .062. Overall, 100 m differed significantly from 200 and 400 m, as well as 200 from 400 m (p<.001) with lower values in longer distances (cf. table 4). For the strokes, there was a significant difference (p = .009) between breaststroke and backstroke, with higher mean values for the backstroke events. For the 100 m events, significant differences were found between freestyle and butterfly (p = .009), as well as between freestyle and breaststroke (p = .001), but only in men. Thereby the values in freestyle were the highest. For both sexes, the mean values were significantly lower (p = <.001 to.03) with increasing distance for the freestyle events. The comparison of max. bLA for each stroke and sex showed significant differences between men and women for 200 m backstroke (p = .041), 200 m breaststroke (p = .001) and for all freestyle events (p = .001, .002 &.002), with constant higher values in men.

### Multi-Level Analysis (MLA)

The Multi-Level Analysis (MLA) for the predictors sex, stroke and distance is significant in each case (p<.001). The calculation of the Level 2-pseudo-R^2^_C_ to check the individual within variation for each predictor showed a reduction of variance for sex [13.3% (IAT), 21.1% (% of PB on IAT) and 17.7% (max. bLA)], for distance [22.5% (IAT), 40.1% (% of PB on IAT) and 22.1% (max. bLA)] and stroke [6.4% (IAT), 1.1% (% of PB on IAT) and 0% (max. bLA)]. Consequently, according to Cohen [35], predominantly medium effect sizes exist for the predictors sex and distance in initial status, whereas only a small effect exists for stroke on IAT. The Level 2-pseudo-R^2^_S_ showed a reduction of the variance of the slopes for sex [1.4% (IAT), 13% (% of PB on IAT) and 28% (max. bLA)], distance [13.1% (IAT), 52.4% (% of PB on IAT) and 32.6% (max. bLA)] and stroke [7.6% (IAT), 10.4% (% of PB on IAT) and 1% (max. bLA)].

The models with all three predictors each (S, D, St) proves a reduction of variance of in initial status of 36.6% for IAT, 60.3% for % of PB on IAT and 39.9% for max. bLA (Level 2-pseudo-R^2^_C_). A reduction of variance was shown for the slopes of 13.6% for IAT, 62.5% for % of PB on IAT and 55.8% for max. bLA (Level 2-pseudo-R^2^_S_).

For IAT (Model 1, cf. table 5) the time intervals between measurements have a significant influence (p = .005) and the athletes showed different change rates over time (β = 7.95E−8, p = .005), measuring a slight decrease (β = 1.19E−4, p<.009). The IAT of the women (men set to 0) are substantially lower (β = −0.47, p<.001), confirming the descriptive results for the 398 data sets (cf. table 3). The factor distance also shows decreasing IAT with increasing length (β = −0.31, p<.001; 100 m set to 0). There is also a significant effect for IAT for stroke (β = −0.14, p<.001; butterfly set to 0), with the highest values for butterfly. The effect of stroke is difficult to interpret, because this variable is not ordinal scaled and consists of more than two strokes.

**Table 5:** Estimates of Fixed Effects of the multi level analysis (MLA, random intercept & random slope) for IAT [mmol*L^−1^], % of PB on IAT and max. bLA [mmol*L^−1^].

Model 1: IAT [mmol*L^−1^]							
Parameter	Estimate	*SE*	*df*	*t*	*p*	95% Confidence Interval	
						Lower Bound	Upper Bound
Intercept	6.35	0.09	644.33	67.96	.000	6.16	6.54
Time*	−1.19E-4	4.44E-5	71.62	−2.67	.009	−2.07E-4	−3.02E-5
Sex	−0.47	0.08	313.60	−6.16	.000	−0.62	−0.32
Distance	−0.31	0.05	841.46	−6.25	.000	−0.40	−0.21
Stroke	−0.14	0.04	728.85	−3.80	.000	−0.21	−0.07
**Model 2: % of PB on IAT**							
Intercept	86.00	0.27	893.37	322.80	.000	85,47	86.52
Time*	−1.07E-3	1.45E-4	79.86	−7.38	.000	−1,36E-3	−7.83E-4
Sex	3.06	0.24	417.17	12.70	.000	2,58	3.53
Distance	3.14	0.13	1419.17	23.69	.000	2,88	3.40
Stroke	−0.23	0.10	1221.69	−2.25	.025	−0,43	−0.03
**Model 3: max. bLA [mmol** * **L** ^−**1**^ **]**							
Intercept	11.49	0.23	787.76	49.41	.000	11.03	11.94
Time*	4.16E-4	8.60E-5	47.06	4.83	.000	2.42E-4	5.89E-4
Sex	−1.70	0.20	368.90	−8.60	.000	−2.10	−1.32
Distance	−1.45	0.12	1115.11	−12.22	.000	−1.69	−1.22
Stroke	−0.19	0.09	973.45	2.07	.039	9.83E-3	0.37

* Time = number of days between the measuring times of the athlete.

For % of PB on IAT (Model 2, cf. table 5) there is a significant influence of the time intervals between measurements (p<.001) and the athletes showed different change rates over time (β = 1.638, p<.001) with a slight decrease (β = 1.08E-3, p<.001). The % of PB on IAT are significantly higher for women (β = 3.06, p<.001; men set to 0). For the factor distance the % of PB on IAT increases with increasing distance (β = 3.14, p<.001; 100 m set to 0). The effect of stroke is also significant (β = −0.23, p = .025; butterfly set to 0).

For max. bLA (Model 3) the different time periods between measurements are not significant (p = .057), but the change rates over time are significant (β = 4.16E−4; P<.001). The results confirm the descriptive results of the 398 data sets. Women (men set to 0) show significantly lower max. bLA (β = 1.71, p<.001). For distance (100 m set to 0) there are lower bLA values for longer distances (β = 1.45, p<.001). The differences of max. bLA for the variable stroke (butterfly set to 0) are significant (p = .039) but slight (β = 0.19).

## Discussion

The present study examined the influence of sex, stroke and distance on the IAT, the % of PB on IAT and the max. bLA in high performance swimming. Furthermore the MLA was used as a method, which is able to analyze typical data structures in high performance sports. Compared to other studies [9], [49] the calculated IAT in this study (range of means: 5.19–7.52 mmol*L^−1^) seem to be very high. Otherwise, Dekerle and Pelayo [50] described, that lactate thresholds in swimming occur at speeds up to 90% of the 200 m pace, which is the case in most events (cf. table 3) in this study. Furthermore, Faude et al. [44] reported higher running speeds/power output in watt at IAT than at MLSS using the threshold concept of Bunc et al. [11]. MLSS concentrations are reported for swimming up to 3–5 mmol*L^−1^ [50]. Therefore the calculated IATs seem to be realistic with the used threshold concept. Furthermore, the differences show that the IAT is strongly dependent on the applied method [1], [50]. Irrespective of the mean values of the IAT, it was not the aim of the study to give recommendations for training intensities on basis of the IAT, but to identify lactate-affecting factors. According to current research, the lack of evidence for the effect of threshold training [51], the positive findings of high intensity training (HIT) on the endurance capacity [52], [53], conceptions like the polarized training model [54] lead to a critical perspective on the “classical” threshold training. The results for the three independent variables are discussed in the following paragraphs.

### Sex

The descriptive data (cf. table 3) and estimations of the MLA (cf. table 5) show in average 0.4–0.6 [mmol*L^−1^] (β = −0.47; p<.001) lower IAT and 1–2 [mmol*L^−1^] lower max. bLA (β = −1.70; p<.001) for women compared to men. These effects are significant, both in ANOVA and MLA, with medium effect sizes. For the IAT these sex specific differences are only significant for 200 m freestyle, whereas the differences for max. bLA are significant for five events (200 m Ba & Br and the freestyle events). These findings are similar to the results of Vescovi et al. [20] where sex specific differences in post-race bLA were only found for the freestyle events. With women reaching lower lactate concentrations in swimming, being in agreement with the findings of for resistance training. Together with muscle mass, the area occupied by muscle fiber types and the size of the fibers types seem to be sex specific, described by for the m. vastus lateralis. This could provide an explanation on a physiological level. With the metabolic characteristics of the muscle fiber types, the connection to the lactate kinetic is given [5], [7], [13]. The overall lower lactate concentrations on IAT and max. bLA in women support the idea of a better developed aerobic metabolism in women compared to men [21], [22]. For % of PB on IAT, both statistical methods showed significant influences for sex (p<.001) with higher values for women (β = 3.06). These differences are also significant between men and women in 7 of 8 events (cf. table 3). The results are supported by, reporting a more economic swimming of women, explaining the lower lactate concentrations and the higher % of PB values at submaximal intensities. Other reasons could be a greater proportion of fatty tissue and different distribution in women compared to men [18]. This can give women a higher net buoyancy [55]. Furthermore, Caspersen et al. [18] reported lower added mass in women as a result of differences of body shape, which seems to be positive for the drag.

### Stroke

For both statistical methods the effects for stroke is significant for all three dependent variables. Although the influence of the stroke is significant in each Model of the MLA (cf. table 5), there are only little reductions of variance for initial status of 0–6,4% (Level 2-pseudo-R^2^_C_) and for the slopes of 1–11,4% (Level 2-pseudo-R^2^_S_), meaning no to small effect sizes for stroke. For the max. bLA, the post-hoc analysis (Bonferroni adjusted) showed only a significant difference (p = .009) between breaststroke and backstroke with higher values for backstroke. These findings support the results of other studies [25], [26] which show no stroke differences/different orders for bLA depending on the distance after maximal swim. Other studies [20], [27] reported the highest max. bLA for butterfly, but none of these studies analyzed the influence of stroke on the IAT. Overall the highest IAT were achieved in 100 m butterfly, which differs significantly from all other strokes (p<.001, Bonferroni adjusted). The MLA confirms this results with the highest IATs in butterfly (β = −0.14; p<.001). There are also the lowest % of PB on IAT for butterfly (β = −0.23; p<.025), which differs significantly from backstroke and freestyle (p<.001, Bonferroni adjusted). A possible explanation could be, that around the IAT swimming speed is not at maximum, the statement of Barbosa et al. [15] could be confirmed in terms of economy, with butterfly showing the slightest swimming economy at lower swimming speeds. However the economy improves with increasing speed [26]. The highest IAT was found for 100 m butterfly, presumably connected with the fact that it seems to be difficult to swim butterfly with low intensities (first steps) [26], because of the high demand of interaction between strength and coordination [30], [56]. On the other hand, the butterfly technique entirely makes high demands for strength, thus low intensities (in %) could be a high individual exposure [30]. Therefore, the glycolytic muscle fibers could be recruited at an early stage [57], [58], operating as main lactate producers when recruited [5], [7], explained by the size principle of motor unit recruitment [58], [59]. However these results have to be interpreted carefully because of the small data sets for butterfly. Anyhow, the statements of Barbosa et al. [15] are not confirmed by the descriptive results of the IAT and the max. bLA for the breaststroke events. The lactate concentrations of the breaststroke events are the lowest on average in direct comparison with the same distance for the other strokes. Regarding the 200 m events, the IATs are in a similar zone for both sexes in each case, although the energy consumption in freestyle and backstroke seems to be lower, because of the lower intracyclic variation of swimming velocity compared to butterfly and breaststroke [16], [28]. Summarized, for the variable stroke the biggest effects occurred for butterfly on the IAT for the 100 m events, with the significantly highest values. Overall the stroke seems not to play a key role in terms of affecting lactate parameters. The knowledge about the economy [16], [28] of the strokes is reflected only partly in our results.

### Distance

For distance all ANOVAs and Models of the MLA were significant (p<.001) for the three independent variables IAT, % of PB on IAT and max. bLA. The MLA show reductions of variance for initial status between 22.1 to 40.1% (Level 2-pseudo-R^2^_C_) and for the slopes between 13.1 to 52.4% (Level 2-pseudo-R^2^_S_), meaning mostly large effect sizes. The descriptive data, the ANOVAs and MLA showed significant decreasing IAT (β = −0.31; p<.001), increasing % of PB on IAT (β = 3.14; p<.001) and decreasing max. bLA (β = −1.45; p<.001) with increasing distance. These general results were confirmed by sex and stroke specific analysis only for the freestyle events (Bonferroni adjusted) in men and women. The results are not surprising, because with increasing distance the aerobic capacity becomes more important [32]. From a practitioner perspective a certain versatility of 100 and 200 or 200 and 400 m seems to be attractive to qualify for the 4*100 m and 4*200 m freestyle relays in international competitions, because five to six places are awarded. For the individual events at most two athletes can qualify for each country, therefore the chances to qualify but also the performance density are much higher in the 100 m and 200 m freestyle events. Although Meckel et al. [39] described that swimming is taking a special position in order to achieve world-class performances in various events with different performance profiles, but priorities in the training content are necessary from current point of view [22], which are reflected in the significant differences in the freestyle events. According to actual knowledge it is known, that signaling pathways of endurance training mainly trigger transcriptional changes, while weight training adaptations are caused by changes in mRNA-translation [41]. Concerning this, it is unclear whether signaling pathways initiated by weight training or endurance training overlap or hinder each other [38], [40], [41]. Therefore it is not completely clarified at the moment what this specifically means for training periodization, thus e.g. to coordinate strength and endurance training over the season in a synergetic way [59] is important to achieve the best performance in the individual best events on the main competition. Therefore it can be supposed that on the one hand the constitutional conditions and anthropometrical and morphological factors [60] influences the “choice” of the individual special event (sprints or longer distances) and on the other hand the individual specificity is tried to be maintained or optimized in the training process. Consequently a sprinter (50–100 m events) will try to maintain the dominance of muscle fiber type II and train in terms of an “optimum” endurance capacity [54]. An “optimum” endurance capacity means that the quality of training, which implies the training intensity and the exercise tolerance, becomes the key factor and not the volume in kilometers [32]. This point of view confirms the positive effects of high intensity training on the endurance capacity [52], [53] and the concept of the polarized-training model (about 75% of the training below threshold intensity, only 5–10% in threshold intensity and 15–20% above) versus the “classical” threshold-training model [54]. This implements avoiding a shift towards type I fibers by excessive endurance training stimuli, which seems to be only partly reversible [33], [34], [61]. Nevertheless, in swimming, current training regimes seem to be characterized by an aerobic predominance [39], which is possibly not up to date anymore for sprint and middle distances.

### Methodical Discussion

The large number of data sets of athletes at highest performance level supports the explanatory power of the results. It is to be mentioned, that the threshold concept of Bunc et al. [11] is not swim-specific. However, the Pansold-test is characterized by a step-shaped load protocol, which is carried out under field conditions. Therefore a reliable realization of the Pansold-test requires a good sense of time of the swimmers with the ability to swim evenly, particularly in the first steps. Short-term speed increases or to quick beginning speeds can lead to high first-step lactate concentrations. This lactate concentration influences the further course of the test and the lactate curve [44]. However this is accepted for the examination of the endurance capacity in the field accounting for the different structures of swimming disciplines. A methodical strength of this study is the statistical analysis with a MLA. This method allows analyzing different numbers of data sets for an athlete with different time intervals between measurements [46]. For elite sport, it seems to be important to consider the time periods between performance diagnostics to make statements about e.g. the effect of training input on the lactate kinetics without excluding data. For that reason it was possible to analyze all 1902 valid data sets in the second part. Furthermore it is common, that some athletes are part of the squad for longer time periods, experiencing more performance diagnostics than other athletes. A disadvantage of this method is, that detailed information about descriptive data cannot be provided because of the different numbers of data sets for each athlete. A limitation of this retrospective analysis of data between 1992–2010 is, that the equipment used for the lactate diagnostics and the exact process of acquisition is unknown.

## Conclusion

In conclusion, we identified the influence, especially of sex and distance, on lactate parameters in swimming and tried to explain them with current physiological knowledge. Furthermore we showed the importance of considering the different time periods between measurements in a formally correct way by using the MLA for general statements on basis of large data sets in high performance sports. The slight but significant influences of the time periods between measurements show the dynamic and sensitivity of the lactate molecule. These findings may help interpreting results of lactate tests in context of e.g. strength parameters or as a consequence of specific training content explained by physiological adaptations (e.g. metabolism of fiber types) or sex specific factors like body density or a better developed aerobic/anaerobic metabolism. Men overall showed higher IAT and max. bLA lactate concentrations compared to women. Whereas in submaximal intensity women achieved higher % of PB with lower lactate concentrations compared to men, which confirms a more economical technique [15], [16] and a better developed aerobic metabolism [22] in women. For the variable stroke, the MLA showed significant results but no or small effect sizes. Therefore, when comparing inter- and intraindividual results of lactate tests in swimming, especially sex and distance specific lactate parameters have to be considered for initial status measurements and the development over time. In particular, longitudinal comparisons under steady conditions, which means applying the same test protocol and threshold concept, could benefit from this [1], [51].
